# Supplementary material for: Psychosocial and pandemic-related circumstances of suicide deaths in 2020: Evidence from the National Violent Death Reporting System
Source: PLoS One. 2024 Oct 11;19(10):e0312027. doi: 10.1371/journal.pone.0312027 (PMC11469549; doi:10.1371/journal.pone.0312027)
Supplement: S1 Fig — (DOCX) [file pone.0312027.s001.docx]

**S7 Figure.** Number of suicides by week in 2020 vs. the pre-pandemic period


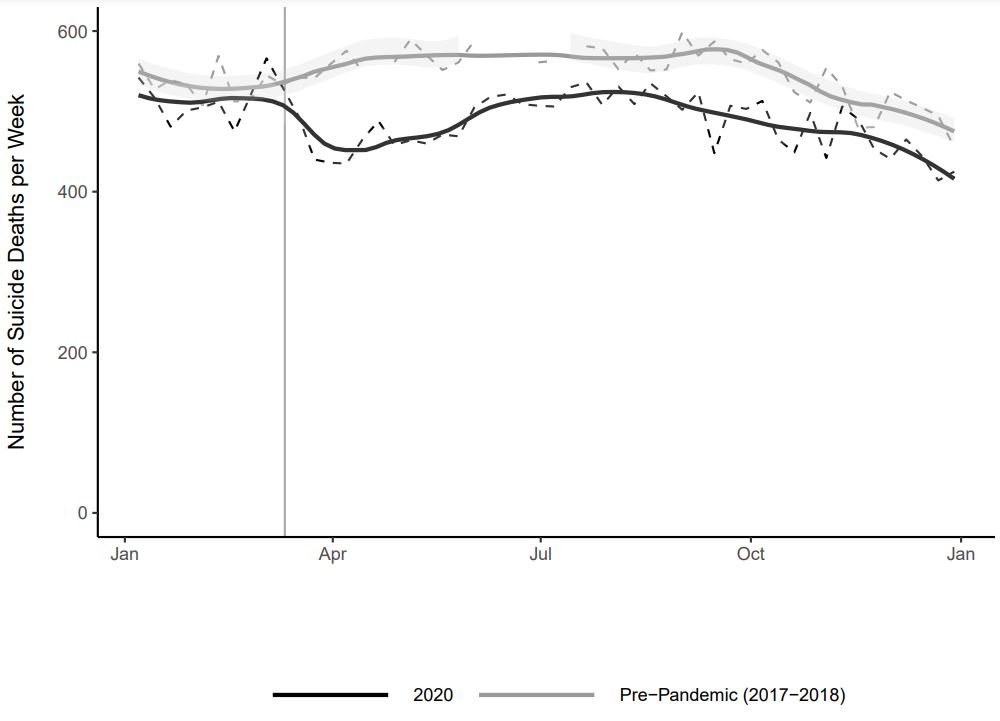


**Caption for S7 Figure:** The x-axis is months of the year. The y-axis is the fraction of suicide deaths in 2020 (n=25,612) relative to the pre-pandemic period 2017-2018 (n=57,484). Values >1 indicate that suicide deaths occurred more frequently in that 4-week period of 2020 vs. the same 4-weeks of the pre-pandemic period. Error bars reflect uncertainty in the pre-pandemic case counts assuming a Poisson distribution.
