# Supplementary material for: Psychosocial and pandemic-related circumstances of suicide deaths in 2020: Evidence from the National Violent Death Reporting System
Source: PLoS One. 2024 Oct 11;19(10):e0312027. doi: 10.1371/journal.pone.0312027 (PMC11469549; doi:10.1371/journal.pone.0312027)
Supplement: S2 Fig — (DOCX) [file pone.0312027.s002.docx]

**S8 Figure.** Sensitivity analysis examining robustness of the time-series analysis to alternative pre-pandemic reference periods


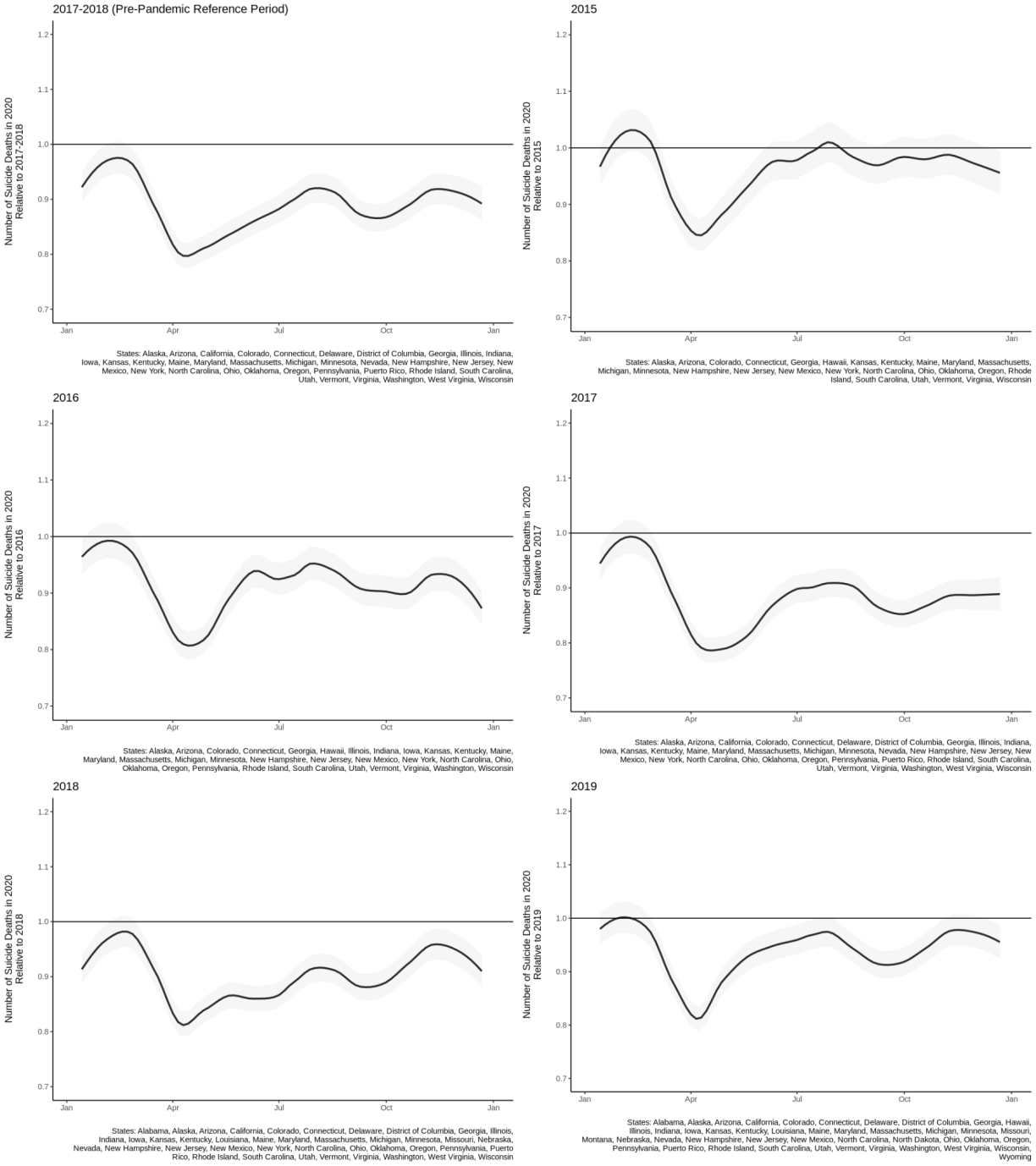


**Caption for S8:** The x-axis represents months of the year. The y-axis represents the number of suicide deaths in 2020 relative to the stated pre-pandemic reference year(s). Since not all states reported into NVDRS in every year, each plot includes only the states reporting during the reference years, and these states are indicated below each figure).
