# Supplementary material for: Psychosocial and pandemic-related circumstances of suicide deaths in 2020: Evidence from the National Violent Death Reporting System
Source: PLoS One. 2024 Oct 11;19(10):e0312027. doi: 10.1371/journal.pone.0312027 (PMC11469549; doi:10.1371/journal.pone.0312027)
Supplement: S3 Fig — (DOCX) [file pone.0312027.s003.docx]

**S9 Figure:** Relative number of deaths in 2020 by whether the case narrative described pandemic-related circumstances (PrC)


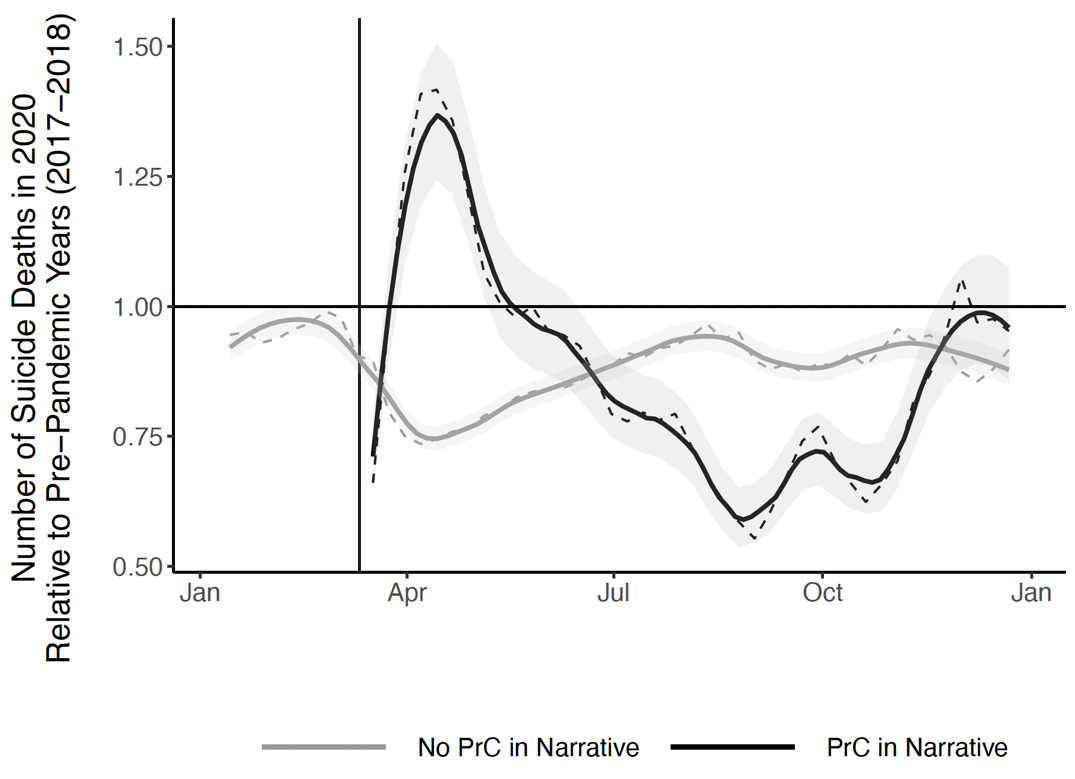


**Caption for S9 Figure:** The x-axis is months of the year. The y-axis is the ratio of suicide deaths in 2020 that did not have PrC (gray line, n=25,578) and the fraction that did have PrC (black line, n=1,909), relative to the pre-pandemic period 2017-2018. Values >1 indicate that the circumstance occurred more frequently in the stated year vs. reference period.
