# Supplementary material for: Psychosocial and pandemic-related circumstances of suicide deaths in 2020: Evidence from the National Violent Death Reporting System
Source: PLoS One. 2024 Oct 11;19(10):e0312027. doi: 10.1371/journal.pone.0312027 (PMC11469549; doi:10.1371/journal.pone.0312027)
Supplement: S4 Fig — (DOCX) [file pone.0312027.s004.docx]

**S10 Figure.** Relationship between demographic characteristics and topic prevalence


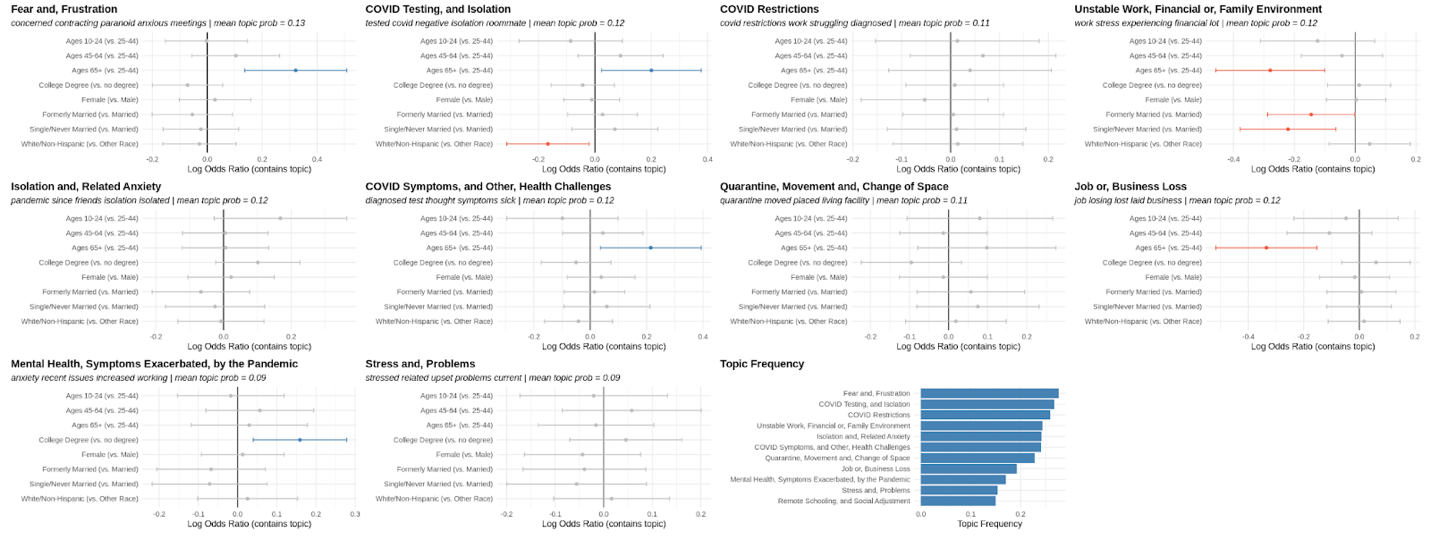


**Caption for S10 Figure:** Explore demographic variation in topic probabilities using a beta regression. 95% confidence intervals for each coefficient are adjusted for multiple comparisons using the Holm-Bonferroni correction. Coefficients are colored in blue if they are significant and positive, in red if they are statistically significant and negative, and in gray if they are not significant.
