# Supplementary material for: Psychosocial and pandemic-related circumstances of suicide deaths in 2020: Evidence from the National Violent Death Reporting System
Source: PLoS One. 2024 Oct 11;19(10):e0312027. doi: 10.1371/journal.pone.0312027 (PMC11469549; doi:10.1371/journal.pone.0312027)
Supplement: S1 Table — (DOCX) [file pone.0312027.s005.docx]

**S4 Table.** Additional sample characteristics during the pre-pandemic (2017-2018) and pandemic (2020) time periods

|  | **Pre-pandemic vs. pandemic time periods** | | | **Year 2020 only** | | |
| --- | --- | --- | --- | --- | --- | --- |
|  | **Years:**  **2017 - 2018**  (n=61,019) | **Year: 2020**  (n=28,001) | **p-value** | **Narratives that did not have PrC** (n=33,359) | **Narratives that had PrC** (n=2,502) | **p-value** |
| **Autopsy Performed** |  |  | <0.001 |  |  | 0.001 |
| Full or Partial | 33507 (55.0%) | 14716 (52.7%) |  | 17007 (51.1%) | 1191 (47.9%) |  |
| Not Autopsied | 27322 (44.9%) | 13062 (46.8%) |  | 16054 (48.3%) | 1289 (51.8%) |  |
| Unknown | 87 (0.1%) | 124 (0.4%) |  | 200 (0.6%) | 9 (0.4%) |  |
| **Means of Injury** |  |  | <0.001 |  |  | <0.001 |
| Firearm | 25439 (41.7%) | 12265 (43.8%) |  | 15318 (45.9%) | 1181 (47.2%) |  |
| Poisoning | 13083 (21.4%) | 5857 (20.9%) |  | 6466 (19.4%) | 368 (14.7%) |  |
| Hanging, strangulation, suffocation | 16818 (27.6%) | 7143 (25.5%) |  | 8456 (25.3%) | 692 (27.7%) |  |
| Sharp or Blunt Instrument | 1288 (2.1%) | 692 (2.5%) |  | 774 (2.3%) | 83 (3.3%) |  |
| Fall | 1644 (2.7%) | 777 (2.8%) |  | 863 (2.6%) | 92 (3.7%) |  |
| Drowning | 753 (1.2%) | 382 (1.4%) |  | 425 (1.3%) | 37 (1.5%) |  |
| Motor or Transport Vehicle | 949 (1.6%) | 479 (1.7%) |  | 572 (1.7%) | 29 (1.2%) |  |
| Other | 593 (1.0%) | 238 (0.8%) |  | 292 (0.9%) | 14 (0.6%) |  |
| Unknown or Missing | 449 (0.7%) | 168 (0.6%) |  | 193 (0.6%) | 6 (0.2%) |  |
| **Disaster Exposure*** |  |  | <0.001 |  |  | <0.001 |
| No, Unknown | 60920 (99.8%) | 26603 (95.0%) |  | 33271 (99.7%) | 973 (38.9%) |  |
| Yes | 99 (0.2%) | 1398 (5.0%) |  | 88 (0.3%) | 1529 (61.1%) |  |
| **Yearly Quarters** |  |  | <0.001 |  |  | <0.001 |
| January - March | 13778 (24.0%) | 6502 (25.7%) |  | 8292 (27.3%) | 167 (7.3%) |  |
| April - June | 15018 (26.2%) | 6243 (24.7%) |  | 7196 (23.7%) | 913 (40.1%) |  |
| July - September | 14974 (26.1%) | 6675 (26.4%) |  | 7992 (26.3%) | 595 (26.1%) |  |
| October - December | 13521 (23.6%) | 5901 (23.3%) |  | 6892 (22.7%) | 601 (26.4%) |  |
| **Narrative Character Length (Mean, SD)** |  |  |  |  |  |  |
| Coroner/Medical Examiner Narratives | 804.0 (514.2) | 853.5 (535.1) | <0.001 | 849.1 (557.0) | 1143.6 (768.4) | <0.001 |
| Law Enforcement Narratives | 836.2 (683.5) | 768.4 (583.0) | <0.001 | 758.6 (584.0) | 980.1 (747.2) | <0.001 |

PrC: Pandemic-related circumstance in the CME/LE narrative.

The variable “Disaster exposure” is an indicator of natural or human-made disasters, which was modified in November 2020 to include problems related to COVID-19. Because of its broad definition and delayed expansion to include the pandemic, it is limited as an indicator of PrC. We therefore only include it in this table to illustrate its distribution in our analytic samples.
