# Supplementary material for: Psychosocial and pandemic-related circumstances of suicide deaths in 2020: Evidence from the National Violent Death Reporting System
Source: PLoS One. 2024 Oct 11;19(10):e0312027. doi: 10.1371/journal.pone.0312027 (PMC11469549; doi:10.1371/journal.pone.0312027)
Supplement: S2 Table — (DOCX) [file pone.0312027.s006.docx]

**S6 Table.** Topic modeling of narratives with pandemic-related circumstances, including exemplar words and phrases.

| **Topic Name** | **5 most**  **common words** | **n (%) of PrC Narratives** | **Excerpts from exemplar narratives** |
| --- | --- | --- | --- |
| Fear and frustration | Concerned; Contracting; Paranoid; Anxious; Meetings | 528 (20.6%) | “...victim's family reported that victim had become extremely paranoid about contracting covid-19 due to the traveling he had to a high-risk area…”  “...victim had recently become paranoid over the coronavirus outbreak and was hoarding food and other supplies prior to death…”  “...the victim had depression due to limited contact with family because of covid…” |
| COVID testing and isolation | Tested; Covid; Negative; Isolation; Roommate | 511 (19.9%) | “...the victim's spouse was in the hospital with covid and victim’s mother tested positive for covid the morning of the incident. The victim disclosed that they did not want to live anymore…”  “...victim had learned that they tested positive for covid today after becoming ill seven days ago…”  “...victim had been to family doctor numerous times, and was tested for covid two separate times, both with negative results…” |
| COVID Restrictions | Covid; Restrictions; Work; Struggling; Diagnosed | 497 (19.3%) | “...victim was not unable to do usual daily activities due to social distancing and covid…”  “...with covid restrictions, victim had resulted in playing more and more video games…”  “...the victim had recently been saddened by social isolation due to covid-19, and was frustrated with their deteriorating hearing and vision…” |
| Unstable work, financial, and/or family environment | Work; Stress; Experiencing; Financial; Lot | 465 (18.1%) | “...victim had suffered from depression and was home for the past two weeks due to their company shutting down as a result of covid-19…”  “...the victim's business had been significantly impacted by the lockdown and the covid-19 pandemic…”  “...the victim wasn't able to work during covid yet the bills continued to come due causing a great deal of financial stress for the victim…” |
| Isolation and related anxiety | Pandemic; Since; Friends; Isolation; Isolated | 462 (18.0%) | “...victim reportedly had become increasingly depressed and complained of being isolated from their friends and significant other during the covid-19 pandemic as they did not go outside of the house for several weeks…”  “...victim feels socially isolated due to the pandemic and that is causing increased depression and anxiety…”  “...the victim was struggling during the covid-19 pandemic and the holidays were especially hard because of not being able to visit their friends and family…” |
| COVID symptoms and other health problems | Diagnosed; Test; Thought; Symptoms; Sick | 460 (17.9%) | “...per sibling, the victim was a hypochondriac and traveled out of country for 2-3 months and believed to have covid-19…”  “...the spouse reported the blackened toes were signs of covid-19 and the virus had begun to infect victim in different ways as well, including loss of appetite…”  “...covid-19 made drug testing less frequent, possibly contributing to a relapse…” |
| Quarantine, movement, and change of space | Quarantine; Moved; Placed; Living; Facility | 435 (16.9%) | “...victim was having a hard time coping with the quarantine because the victim couldn't go see the victim 's friends or go golfing, which victim did daily…”  “...victim had moved to an assisted living facility x months prior and had been isolated for the past x months due to covid…”  “...parent stated that the victim had recently been down due to being isolated at home during the covid-19 pandemic…” |
| Job or business loss | Job; Losing; Lost; Laid; Business | 367 (14.3%) | “...victim was concerned about their business being closed due to the covid-19 pandemic…”  “...while in quarantine the victim was reportedly worried that they were going to lose their job…”  “...​​victim recently lost their job due to the pandemic, and were receiving unemployment benefits…”  “...victim 's friend reported that the victim was depressed because of a recent job loss and income due to the covid-19 business closures and had been expressing suicidal thoughts…” |
| Mental health symptoms exacerbated by the pandemic | Anxiety; Recent; Issues; Increased; Working | 327 (12.7%) | “...victim's parent stated that stress from the covid-19 situation spurred most of victim 's recent anxiety and stress…”  “...the friend also mentions the victim had anxiety issues that were exacerbated by the coronavirus pandemic…”  “...victims spouse related that victim had been more depressed than usual as they were concerned about the current events surrounding the covid-19 outbreak along with a series of recent deaths in the family…” |
| Stress and problems | Stressed; Related; Upset; Problems; Current | 295 (11.5%) | “...victim was possibly exposed to covid-19 and was upset that they were required to quarantine…”  “...victim had been recently estranged from family and was stressed because of inability to attend family gatherings due to the covid-19 pandemic…”  “...the victim 's sibling advised the victim was depressed with the news and the current situation related to covid-19…” |
| Remote schooling and social adjustment | School; Social; Learning; Online; Struggling | 287 (11.2%) | “...victim was an educator and was notified of extended school closures due to covid-19…”  “...the victim was struggling with covid restrictions and how it would affect their senior year of high school…”  “...due to covid-19, victim attended online school and was frustrated and procrastinated working on school assignments…” |
| The frequences and percentages in this table refer to the number and proportion of narratives containing a representation of each topic above the topic probability threshold of 0.1, as detailed in S3 Appendix . In accordance with the requirements of the NVDRS data sharing agreement, all cited narratives have been modified to protect the privacy of decedents. | | | |
