# Supplementary material for: Psychosocial and pandemic-related circumstances of suicide deaths in 2020: Evidence from the National Violent Death Reporting System
Source: PLoS One. 2024 Oct 11;19(10):e0312027. doi: 10.1371/journal.pone.0312027 (PMC11469549; doi:10.1371/journal.pone.0312027)
Supplement: S1 Appendix — (DOCX) [file pone.0312027.s007.docx]

**S1 Appendix.** Methods: Details regarding NVDRS quantitative variables used in this analysis

**1. Demographic characteristics**

These quantitative variables included: age group [coded as 10-24, 25-44, 45-64, and 65 and older]; sex [male, female]; race/ethnicity [non-Hispanic white, American Indian/Alaska Native, Asian/Pacific Islander, Black/African American, Hispanic/Latino, and Multiracial/Other]; education [≤8th grade, 9-12th grade, diploma/GED, some college, associate’s degree, bachelor’s degree, master’s degree, doctorate degree]; relationship status [married/partnered, single/never married, and widowed/divorced/separated]; date of death (or, if unavailable, date of injury); autopsy status [full/partial vs. no autopsy]; and means of injury [firearm, poison, hanging/suffocation, sharp/blunt instrument, fall, drowning, vehicle, other]. Finally, because the existing NVDRS quantitative variables provide only limited information on employment characteristics, we created a 6-level categorical indicator for the labor force status using a textual keyword search of the text fields “Occupation Current Text” and “Occupation Text_DC”. This variable was coded as: industry employment, self-employed, disability, retirement, student, unemployment, which incorporated decedents who were incarcerated, and unknown/missing.

**2. Quantitative Indicators for the presence of other “Contributing Circumstances”**

NVDRS data includes a range of binary “circumstance variables” that encode precipitating key events or behaviors that were flagged as noteworthy in source documents, each coded as *‘Yes vs. No/Not Available/Unknown.’* NVDRS abstractors are instructed to code a circumstance when: a) it signifies a problem around mental health, alcohol or substance abuse, and b) a circumstance is not mental or substance use problem, but is perceived it to be relevant to death and/or occurs in a close temporal proximity to a death event.

Circumstance variables also include indicators for whether a particular event or behavior has been acute or a crisis, which aims to identify an element of impulsivity in the decedent's behavior. For purposes of identifying and assessing contextual risk factors of violent deaths, NVDRS circumstance variables encode valuable information, however these variables are often limited by data availability and structural changes in data processing. For example, a recent validation study of Oregon NVDRS behavioral variables found that these circumstance variables missed to indicate about a half of cases with known mental health and substance use problems.^1^

To operationalize correlates of suicide risk, we created dichotomous summary indicators of the following seven circumstance variables (each coded Any vs. None). Among youth ages 10-24, we examine 1) *School problems*. Among adults ages 25+, we examine the following correlates: 2) *Socioeconomic problems* (i.e., financial problem, job problem, or eviction or loss of housing), 3) *Mental health* (i.e., mental health problem, or history of suicide ideation, intent or attempt), 4) *Substance abuse* (i.e., alcohol problem, substance abuse, or other addiction), 5) *Physical health problems*, 6) *Interpersonal problems* (i.e., problems with intimate partner, family relationship, or other relationships, or argument), and 7) *Bereavement* (i.e., recent death of family member or friend). Confirmatory factor analysis suggests that these constructs are a reasonable fit to the data (comparative fit index = 0.87).

**3. Missing data.**

Since the primary focus of our analysis was to describe precipitating circumstances of suicide mortality during the pandemic, all our analyses were restricted to cases with known circumstances, which are indexed through the key gateway variable “Circumstances Known” in the NVDRS data. For the 2020 data (see **Figure 1**), 7285 (20%) cases had no known circumstances. After restricting our data to known circumstances, less than 1% of missingness was present in the NVDRS-coded circumstance variables. Compared to decedents with no known circumstances, decedents included in the study were more often in their early- to -middle-adulthood, white, with higher education level, less often married, more often employed, and more likely to have died due to poisoning or hanging/suffocation.

Citation:

1. Carlson, K. F., Gilbert, T. A., DeFrancesco, S., Wright, D. A., Shen, X., & Cook, L. J. (2022). Accuracy of behavioral health variables in Oregon national violent death reporting system data: a linked cohort study. Injury epidemiology, 9(1), 29.
