# Supplementary material for: Psychosocial and pandemic-related circumstances of suicide deaths in 2020: Evidence from the National Violent Death Reporting System
Source: PLoS One. 2024 Oct 11;19(10):e0312027. doi: 10.1371/journal.pone.0312027 (PMC11469549; doi:10.1371/journal.pone.0312027)

**S2 Appendix. Methods:** Details regarding identification of Pandemic-related Circumstances (PrC) from the text narratives.

**1. Identification of Cases with Pandemic-Related Circumstances (PrC).**

Since CME (n=34,816, median: 744, min-max: 3-11,936) and LE (n=29,433, median: 665, min-max: 1-10,240) reports encode complementary, but distinct contextual information, this study used joint textual data from both CME and LE narratives (n=35,861, median: 1286, min-max: 47-19,549) to identify cases that explicitly referenced COVID-19 pandemic as a significant or notable death circumstance.

1. **Inclusion using keywords search and word embeddings.** First, we parsed the coroner/medical examiner (CME) narrative texts using a set of 21 regular expressions (i.e., “covid”, “pandemic”, “COV-19” ). We improved our set of keywords with a *word2vec* word embedding algorithm that, by calculating semantic similarity between COVID-related words, identified alternative terms used to describe the impact of pandemic in the narratives[^24^](https://www.zotero.org/google-docs/?xNjNo9) (“quarantine,” “isolation”, “lockdowns,” “distance learning”, “shelter-in-place orders”).
2. **Exclusions based on keywords.** After a review of a random subset of narratives, we identified a set of exclusion phrases and keywords, which indicated whether the decedent had a negative COVID test or infection (e.g., “covid: negative”, “covid: no”). However, those narratives that referenced BOTH: 1) whether a decedent had a negative COVID test, and 2) a clear indication that COVID-related circumstances had been a source of distress or a salient feature (e.g., if a narrative said both “victim struggled with distance learning” and “covid: negative”), were indexed as true positive cases.
3. **Disaster Indicator.** We used the disaster exposure variable to identify 94 (0.3%) narratives not captured using keywords. After reviewing these cases manually, we added 6 narratives that encode an association between death and pandemic using phrases that explicitly reference the pandemic using idioms or terms that were not synonymous with our original inclusion phrases (i.e., “current state of the world”, “e-learning”).
4. **Manual review of COVID positive/negative and stay-at-home cases.** Finally, through several iterations of manual reviews, we added 20 new narratives, but removed a subset of narratives (77 (0.2%)) with a reference to COVID testing only (e.g., “tested positive for COVID”), but no explicit indication that pandemic was rendered as a significant death circumstance. The annotated cases were then added to the rest of the sample, yielding a final analytic sample of 2502 cases.
5. **Manual review without addition of cases.** We did not include “isolation” as part of the keywords searches because the inclusion of this term alone produced a high number of false positive cases. However, to ensure we did not have false negative cases, we conducted a manual review of 125 cases that contained a regular expression “isolat”, but were indexed as a non-case by the previous classification criteria. This suggests that, when referencing feelings of isolation caused by pandemic shutdowns, the phrase “isolat*” is combined with other keywords in our list. An example of a true negative case with a term “isolating”:

*”Next of kin advised the police that V had been under a lot of stress lately due to family issues and had been isolating himself from everyone.”*

Finally, our check confirmed that “isolation” and derivative terms were showing up as frequently in the previous years as they were in narratives with no reference to COVID in 2020. Overall, our approach identified 210 narratives containing “isolat*” that reference pandemic and 207 that do not.

**2. Validation of our approach to identifying cases with PrC.**

Our approach toward identification of PrC narratives was validated by 3 independent raters through manual annotation of a subset of 250 PrC-positive and 250 PrC-negative narratives, randomly selected from the final sample. Interrater agreement was calculated using a sample of 50 PrC-positive and 50 PrC-negative cases that all three raters independently labeled. The annotators initially had high inter-annotator agreement (Krippendorff's alpha = 0.81), which was improved by group discussion of discordant labels and case definition (final Krippendorff’s alpha=0.90).

Below we provide descriptions, excerpts from the narratives, and final ‘assignment’ of illustrative discordant labels from this sample of 100:

- **Situations in which circumstances such as job loss occurred during the acute phase of the pandemic but were not explicitly linked to the pandemic in the text.** For example, “Decedent used to be a paramedic and had more recently worked as a medical courier. V. had been terminated from their position several weeks ago.” This case was labeled as *PrC-negative* after discussion among the three independent raters.
- **Situations that occurred as secondary, reverberating impacts of the pandemic.** For example, “Decedent was in a lot of pain 2 days prior to [...] death and always talked about how much pain [...] was in. The V couldn't get [...] lupus prescription because of the President's claim on Lupus medications in regards to COVID-19.” This case was labeled as *PrC-positive* after discussion among the raters.
- **Positive COVID tests/treatment for COVID (which was typically used as an exclusion phrase) that resulted in distress.** For example, “[...] was discharged from the hospital with medications (Eliquis and hydrocodone) due to concerns of having COVID-19. [...] was reportedly in a lot of pain and did not want to be discharged from the hospital.”. This case was labeled as *PrC-positive* after discussion among the raters.

These unclear cases were discussed, and the case definition guidelines were amended in order to clarify that, for a narrative to be labeled as ‘PrC-positive,’ it had to contain some circumstance that could be understood as both a) a stressor and b) pandemic-related. To satisfy a), it is sufficient if the circumstance is mentioned in the narrative, as long as the narrative doesn’t indicate that the circumstance was *not* a stressor or was uncertain (e.g., “V was excited to do remote schooling” or “V hadn’t received his stimulus check yet but it is unknown if this caused any stress.”). In short, the circumstance, event, or stressor had to be explicitly pandemic-related but did not have to be the key driver for suicide-related behavior. To satisfy b), the circumstance had to be pandemic-related using one of the criteria given in the original definition.

Finally, reviewers were asked to independently update their scores in light of these new guidelines, from which the post-discussion inter annotator agreement score was calculated (updated alpha=0.90). Given the high agreement, the remaining cases were each labeled by two annotators to allow for calculation of positive and negative predictive value.

**3. Calculation of Positive Predictive Value (PPV) of the PrC case identification approach:** To help us gauge the extent to which we were able to correctly classify cases that contain pandemic-related circumstances as PrC (i.e., to calculate PPV), two authors independently manually labeled a random sample of 250 PrC-positive cases. Among this 250, there were 8 instances where authors initially disagreed on their assessments; all discordant labels were resolved through discussion until there was a final agreed-upon adjudication of all cases. Each author found 19 instances that don’t contain pandemic-related circumstances (i.e., 19 false positives), for a PPV of 0.92.

**4. Calculation of Negative predictive value (NPV) of the PrC case identification approach:** To help us gauge the extent to which we were able to correctly classify cases that do not explicitly contain pandemic-related circumstances as non-PrC (i.e., to calculate NPV), we manually labeled a random draw of 250 cases from the entire non-PrC subsample. None of these 250 cases contained pandemic-related circumstances (i.e., 0 false negatives).

Additional subsamples to calculate NPV. Since the prevalence of PrC cases is expected to be low, the likelihood of seeing 0 false negatives in 250 cases is non-trivial -- even if the true NPV of the procedure is not 1. Since COVID-19 mentions are relatively low-frequency, the false negative rate will likely be underestimated by labeling a random sample of all narratives. To verify that we have not missed a large number of PrC cases, we conduct further validation by calculating the false negative rates among two subsets of PrC negative cases that are more likely to contain false negatives:

1. **Subsample of ‘PrC-negative’ cases (n=100) selected based on keyword phrase similarity in the narratives:** We identify 600 narratives containing noun and verb phrases that are semantically similar to phrases that describe pandemic impacts in PrC cases. We 1) used spaCy’s dependency parser to extract noun and verb headed subtrees from sentences in the 2020 non-PrC narratives, and 2) compared the Sentence-BERT embeddings from the sentence-transformers library^2^ of the target phrases to the embeddings of the noun and verb phrases in the remaining narratives. Specifically, a phrase is semantically similar if its Sentence-BERT embedding has cosine similarity at least 0.7 to one of the following target phrases: covid, covid-19, pandemic, sars cov-2, quarantine, isolation, distance learning, remote schooling, state of the world (e.g., ‘the corona-virus’ is similar to 'COVID-19', ‘a bout with the virus’ is similar to 'pandemic', ‘trying to teach from home’ and ‘working remotely’ is similar to 'distance learning', ‘everything happening in the news’ is similar to 'state of the world'). To ensure phrases in this subset are sufficiently similar to the keyphrases, the threshold of 0.7 was determined by examining the empirical distribution of similarity scores and determining a value at which the phrases seem to decrease in quality. Three authors reviewed a random sample of 100 narratives containing these phrases and found one false negative case (“V had written notes to his friends & expressed his thoughts how overwhelming world events currently are”). Each case was reviewed by two authors; the only disagreement was about this one false negative case, which the authors agreed was a false negative after adjudication.
2. **Subsample of ‘PrC-negative’ cases (n=100) selected because they had several characteristics associated with PrC in the “true” PrC-positive cases.** We also calculated the false-negative rate among 152  decedents whose personal characteristics indicate that they have a high likelihood of having PrC. For instance, PrC are more than twice as common among decedents with Financial Problems, in deaths between April and May 2020, and cases with above-median narrative length – and 4.75 times as likely to occur among decedents with all three of these characteristics. We label a random sample of 100 cases with all three of these PrC-associated conditions and found one false negative case (“V was also concerned about getting a stimulus check payment from the government”). Each case was reviewed by two authors; the only disagreement was about this one false negative case, which the authors agreed was a false negative after adjudication.

In total, two of these 200 cases contained pandemic-related circumstances (i.e., 2 false negatives), resulting in a NPV of 0.99. This suggests that we have likely not overestimated NPV, since our confidence interval captures the NPV in a subset of cases that are most likely to contain false negatives.

*Bayesian Estimation of NPV:* Finally, we used a Bayesian model to calculate the expected distribution of the NPV of the PrC identification procedure. Let r be the false negative rate among the PrC negative cases and k be the number of false negative samples found among a random sample of n PrC negative cases. In our case, we labeled 250 PrC negative cases and found 0 false negatives. Therefore, we estimate the distribution of r given n=250 and k=0 using a simulation with rejection sampling, as follows:

Initialize an empty vector R. Repeat the following procedure 1,000,000 times:

1. Randomly sample 1 draw of r from some prior distribution
2. Randomly sample 1 draw of k from a Binomial(n=250, p=r) distribution
3. Reject r if k is not equal to 0; otherwise, add r to R

R represents a random sample from the distribution of r given n=250 and k=0. Therefore, we compute the mean and 95% confidence interval of the entries of R.

However, the estimates depend on the choice of prior distribution for r. We estimate NPV from six prior distributions and report the most conservative estimates. Since r is a rate, it must be in [0,1], so we use uniform and beta prior distributions. To set the parameters of the prior distributions, we assume that there are unlikely more false negatives than true positives and therefore make the conservative assumption that the means of these distributions is =# True Positive Cases# PrC Negative Cases0.076. With this assumption, we test four priors: a uniform distribution over the interval [0,2]=[0,0.15] and three beta distributions with different values of parameter alpha that allow the distribution to be relatively right-skewed (1, 2, and 3) and parameter beta fit such that the mean of the distribution is (12, 24, and 36, respectively).  The Beta(3,36) distribution gives us the most conservative estimate of NPV (expected NPV of 0.99, 95% CI: 0.975, 0.998), which we report in the main paper.

The prior distributions of r are visualized in the plot below.


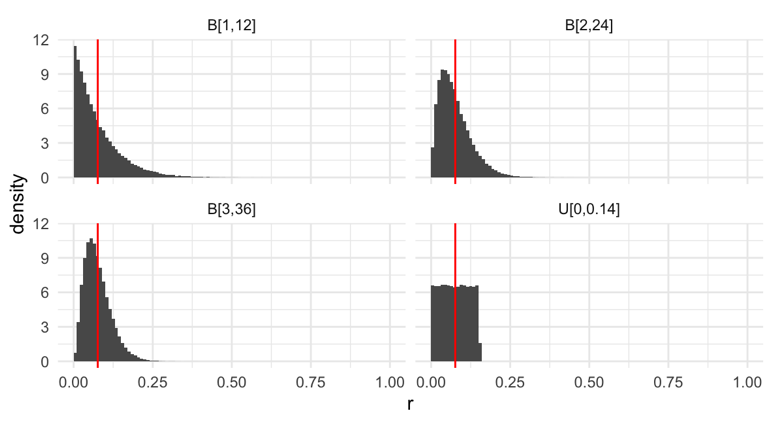


Point estimates and 95% CIs of the NPV from these different r distributions are shown below.


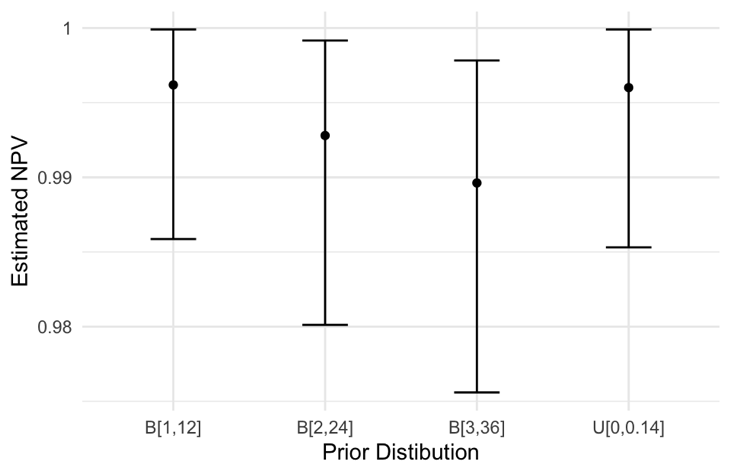

Supplement: S2 Appendix — (DOCX) [file pone.0312027.s008.docx]
