# Supplementary material for: Psychosocial and pandemic-related circumstances of suicide deaths in 2020: Evidence from the National Violent Death Reporting System
Source: PLoS One. 2024 Oct 11;19(10):e0312027. doi: 10.1371/journal.pone.0312027 (PMC11469549; doi:10.1371/journal.pone.0312027)
Supplement: S3 Appendix — (DOCX) [file pone.0312027.s009.docx]

**S3 Appendix.** Methods: Time-series analysis and visualization of suicide deaths over the 12 months of 2020

**Quantifying Uncertainty in Comparisons in the Time-Series Analysis.** Since NVDRS represents a complete suicide mortality registry, we do not have uncertainty in our estimates that is attributable to sampling variability. There is expected annual variability in suicide case counts, reflecting some amount of non-determinacy in the precipitating circumstances and in the actual deaths. We assume monthly case counts were Poisson-distributed, with time-varying means drawn from the 2020 case counts. Using this assumption, we plot the 95% confidence interval of the case counts in each month.

To create these visualizations, we used the following procedure: (a) calculate n_i_ = total cases in month i; (b) calculate c_i_ = total circumstance-related cases in month i; (c) draw n_i_^sim^ = 10,000 values from a Poisson distribution with mean n_i_ and c_i_^sim^ = 10,000 values from a Poisson distribution with mean c_i_; (d) calculate the L_i_ = 2.5^th^ quantile of the c_i_^sim^/n_i_^sim^ distribution and U_i_ = 97.5^th^ quantile of this distribution; and (e) plot c_i_ / n_i_ as the main line with error bars from L_i_ to U_i_. We apply this procedure separately for cases that reference pandemic-related circumstances and those that do not.

**Citation**: Cleveland WS, Devlin SJ. Locally weighted regression: an approach to regression analysis by local fitting. *J Am Stat Assoc*. 1988;83(403):596-610
