# Supplementary material for: Psychosocial and pandemic-related circumstances of suicide deaths in 2020: Evidence from the National Violent Death Reporting System
Source: PLoS One. 2024 Oct 11;19(10):e0312027. doi: 10.1371/journal.pone.0312027 (PMC11469549; doi:10.1371/journal.pone.0312027)
Supplement: S4 Appendix — (DOCX) [file pone.0312027.s010.docx]

**S5 Appendix.** Methods: Topic modeling of the narrative texts that had pandemic-related circumstances

We use NVDRS PrC case narratives to understand the effects of the pandemic on decedents. In order to describe how the pandemic was represented in narratives, we identified 11 frequently discussed themes by taking the following steps:

**1. Cleaning Narrative Text.** In order to capture just the circumstances most directly related to the pandemic, we examine what was described in the sentence(s) of the narrative that mention the pandemic.^[[1]](#footnote-1)^

We split the narrative into sentences using the NLTK PUNKT sentence tokenizer, and identify sentences containing COVID-19 mentions by a) searching for our keywords and phrases in each sentence, and 2) for narratives that did not contain any keywords but were included based on manual review, annotators manually identified the COVID-related sentence(s). Since there can be more than one sentence mentioning COVID per case (e.g., one sentence in the LE and one in the CME), in total we identified 3,813 sentences mentioning COVID, coming from 2,502 out of the 35,861 suicides in 2020.

Downstream analysis required both the full sentence as well as its bag of words representation to determine and assign topics. In order to create the needed features, the COVID-related sentences underwent two rounds of pre-processing. The first round entailed minimal cleaning for readability: turning all text into lowercase (to standardize narratives, since capitalization is often inconsistent), removing non-unicode special characters that cause errors in text processing programs (e.g., the é in fiancée), and expanding common abbreviations in NVDRS that are not common in other settings and, therefore, may not be interpreted appropriately by language models trained on large corpora (e.g., ‘v’ for ‘victim,’ ‘wm’ for ‘white male’). We will call sentences after this first round of pre-processing *cleaned sentences*.

**2. Bag of Words Representation.** Then a second round of pre-processing turned the sentence into a ‘bag of words,’ a term from linguistics that refers to a list of terms contained in the sentence that signal the content of that sentence. In order to carry a useful signal, the bag of words representation must include terms that are a) infrequent enough that their presence in a sentence is meaningful (e.g., a word like ‘the’ would not be any indicator of what a sentence is about, since it is used in most sentences; in suicide narratives frequently used words like ‘victim’ may suffer from the same problems); b) frequent enough to be able to detect patterns in its co-occurrence with other words; and c) semantically meaningful. Therefore, the vocabulary of our bag of words representation consists of all words in the COVID-related sentences that are not: a) common words in the English language (taken from NLTK’s list of stop words, augmented by personal pronouns and prepositions) or in NVDRS narratives (operationalized as the top 200 words, including stopwords but also domain-specific terminology like ‘suicide,’ ‘death,’ ‘victim’); b) words used rarely (i.e,. fewer than 15 times across all sentences); or c) tokens in the text that likely do not correspond to semantically meaningful terms (e.g., words containing 2 or fewer characters, words containing numbers). The second round of pre-processing involved splitting sentences into a list of words using NLTK’s TreeBank word tokenizer^4^ and eliminating words from the list that matched at least one of these four criteria. We will call sentences after the second round of pre-processing a *bag of words*.

We identified 5,934 unique words present across all COVID-relevant sentences, of which 251 words met the criteria for the bag of words’ vocabulary. 310 of the 3,813 (8.1%) COVID-related sentences did not contain any words in this vocabulary, so these sentences were excluded from further analysis.

**3. Unsupervised Topic Model.** Using the cleaned sentences and corresponding ‘bag of words’ representations from (I) (n=3,503), we trained a contextualized topic model in order to identify themes appearing in the text. The model we used, known as the Combined Topic Model in the contextualized_topic_model Python package,^5^ applies 1) large language models to construct contextualized vector representations of and detect semantic similarities between the cleaned sentences and 2) uses the bag of words representation and list of in-vocabulary terms to augment the representation and improve the coherence of the topics (i.e., the extent to which the topic’s top words and associated sentences are related to each other). Using these two features, the Combined Topic Model a) creates a set of topics, each characterized by a set of in-vocabulary terms that are highly associated with that topic, and b) assigns each document a probability distribution over the topics. Each document may load on zero topics (i.e., if it’s assigned low probability to all topics), one topic, or more than one topic (e.g., if there’s a topic related to fear and job loss, we might expect to assign high probability to these two topics.

Through a combination of automated manual review, we selected among several model configurations and parameters. We vary the number of topics (12-20), vocabulary size (removing top 100/200/500 words from NVDRS; removing words with under 10/15/25 used), and the underlying language model used (all-mpnet, paraphrase-distilroberta; the differences include what types of data the model was trained with and how many weights/parameters it has), training three topic models at each parameter set to capture average performance. We calculated the model’s coherence (how similar are items within a topic) and topic diversity (how different are topics from each other) and identified 15 sets of parameters where both of these values were relatively high. Then we manually reviewed the top words and ~15 sentences loading on each topic with probability >0.1, and selected a parameter set that produced largely coherent, interpretable topics (18 topics, 251-word vocabulary, all-mpnet language model).

**4. Supervised Topic Model.** Starting with the best parameters of the unsupervised topic model, a second round of manual review was used to refine the topics. For each of the 18 topics from (II), we reviewed the top words and ~15 sentences loading on each topic with probability >0.1 and created a provisional name and working description of the topic. Starting from these 18 original topics, we identified 3 topics to remove from the model (as they were incoherent or loaded on nonspecific attributes like use of negative valence language) and 5 topics to combine into other topics (as they loaded on similar attributes). We also reclassified school-related sentences, which were split between two topics along with other sentences, into their own topic using keyword heuristics and manual review. This process left us with 11 new heuristic topics. We relabeled each document with these updated topics, and also manually reviewed a set of 10 documents per topic and reclassified them into the appropriate topic(s) as needed (roughly 20% of documents needed to be relabeled).

In order to improve the original topic model, we trained a supervised topic model with the weak labels of the 11 topics generated from our second round of review. We used the implementation of Card et al. (2018)’s work in the SuperCTM function in the contextualized_topic_model Python package.^6^ Like other topic models, this supervised topic model groups semantically and lexically similar sentences and, as such, produces output descriptive of the natural variation in the COVID-related sentences; however, it loosely aligns its output to the labels it was given, in order to improve the relevance of the topics. This second topic model produced 11 topics that were largely, but not entirely, similar to the 11 heuristic topics serving as the original labels. For instance, our heuristic topics included three separate topics relating to stress and anxiety (stress about world events, stress about the getting COVID-19, other stress about/as a result of pandemic), but the final topics had combined these into two, somewhat conceptually different, topics (stress and problems, fear and frustration).

We performed steps (II) and (III) three separate times, in order to obtain different versions of the topic model; the goal was to check the consistency of our approach and be able to select the model producing the best topics.

**5. Finalize Topics.** Finally, two authors selected and finalized the topics for analysis. First, these annotators reviewed the predicted topics and topic distributions from three instances of the model in (III), selecting the model with the best coherence and interpretability. All three models generated the same final named topics, and we selected the version of the model where the sentences loading on each topic seemed most coherent to the human annotators. Then, with the final topic model, the annotators reviewed the top 5 words loading on each topic, 30-50 randomly selected sentences that loaded on each topic (probability of the topic is at least 0.1), and the top ~20 sentences loading most highly on each topic. Using this information, the annotators created names for the topics that encapsulated the terms and documents it subsumes, and selected examples that captured common variation in the topic.

**6. Quantifying Uncertainty in Comparisons in the Topic Modeling Analysis.**

For Figure 4, we are comparing the frequency of topic mentions across decedents of different age groups (rather than comparing case counts per say). We wanted to give some sense of the amount of uncertainty in assigning the 11 topics we identified. Note that we set a threshold of topic probability of >0.10 (at least 10%) for this analysis because this threshold captured most (85%) of the topics represented in the narratives and provided interpretable, relatively homogenous topic groupings (see S6 Table). However, other investigators could have chosen a different topic probability threshold.

Therefore, to generate the error bars in Figure 4, we assume that the number of deaths with PrC is fixed and then model uncertainty in the presence (and assignment) of the 11 topics to the PrC narrative texts using a binomial distribution. Using this approach, comparing two age groups is equivalent to a two-sample proportion test and error bars are taken from a one-sample proportion test.

**7. Analyzing Demographic Variation in Topics.**

To explore demographic variation in topics, we regress the topic probability on age, race, sex, marital status, and education level using a beta regression. Coefficients in this regression represent changes in log odds of the topic, associated with different demographic attributes. 95% confidence intervals for each coefficient are adjusted for multiple comparisons using the Holm-Bonferroni correction. **S10 Figure** plots the regression coefficients; coefficients are colored in blue if they are significant and positive, in red if they are statistically significant and negative, and in gray if they are not significant. We find that there are significant differences across age groups, in many topics, with fewer differences in other demographic categories like race, marital status, and education.

**Citations**

1. Sentence Transformers. Updated 2022. Accessed February 26, 2023. <https://www.sbert.net/>
2. Documentation. nltk.tokenize.punkt module. Updated January 2, 2023. Accessed February 26, 2023. <https://www.nltk.org/api/nltk.tokenize.punkt.html>
3. Documentation. nltk.tokenize.word_tokenize. Updated January 2, 2023. Accessed February 26, 2023. <https://www.nltk.org/api/nltk.tokenize.word_tokenize.html>
4. CombinedTM: Coherent Topic Models. Updated 2020. Accessed February 26, 2023. <https://contextualized-topic-models.readthedocs.io/en/latest/combined.html>
5. Extensions: SuperCTM and β-CTM. Updated 2020. Accessed February 26, 2023. <https://contextualized-topic-models.readthedocs.io/en/latest/extensions.html>

1. Per our direct correspondence with them, NVDRS abstractors often write about each circumstance in its own sentence. [↑](#footnote-ref-1)
