# Supplementary material for: Psychosocial and pandemic-related circumstances of suicide deaths in 2020: Evidence from the National Violent Death Reporting System
Source: PLoS One. 2024 Oct 11;19(10):e0312027. doi: 10.1371/journal.pone.0312027 (PMC11469549; doi:10.1371/journal.pone.0312027)
Supplement: Attachment — Submitted filename: Response to Reviewers 7.17.24.docx [file pone.0312027.s011.docx]

**Comments regarding formatting**

1. **Please note that funding information should not appear in the Acknowledgments section or other areas of your manuscript. We will only publish funding information present in the Funding Statement section of the online submission form. Please remove any funding-related text from the manuscript and let us know how you would like to update your Funding Statement.**

I have removed the funding-related text from the manuscript, as instructed.

The Funding Statement should read as follows:

"This project was supported by grants from the National Institute of Mental Health (R01-MH128198, to MPIs Mezuk and Zivin) and the American Foundation for Suicide Prevention (DIG-1-110-19, to Mezuk). The funders had no role in the conceptualization, analysis, interpretation, or decision to publish this manuscript."

2. **If there are ethical or legal restrictions on sharing a de-identified data set, please explain them in detail (e.g., data contain potentially identifying or sensitive patient information, data are owned by a third-party organization, etc.) and who has imposed them (e.g., a Research Ethics Committee or Institutional Review Board, etc.). Please also provide contact information for a data access committee, ethics committee, or other institutional body to which data requests may be sent.**

The restricted-access data used in this analysis are owned by the CDC’s National Violent Death Reporting System (NVDRS). In order to access these data, we had to agree to the NVDRS’s Data Use Agreement (DUA), which states in Section IIIc (bolded text for emphasis): “The principal investigator(s) and all collaborators agree to employ all reasonable efforts to maintain the confidentiality of the data, with such efforts to be no less than the degree of care to preserve and safeguard its own data. **The principal investigator(s) and all collaborators further agree not to disclose, reveal, or give the data, with or without charge, to any entity or any individual not listed in section III.k.** [this is our list of co-investigators and research staff] **without prior approval from the CDC.** In the event that the principal investigator is required by judicial or administrative process to disclose the data, the principal investigator must: (1) immediately notify the CDC’s National Center for Injury Prevention and Control (NCIPC) and allow CDC a reasonable time to oppose the process; and (2) work in collaboration with the CDC to maintain confidentiality of the data.”

**Therefore, we have provided this Data Availability Statement:** “Data cannot be shared publicly because of legal and ethical considerations to ensure confidentiality. Data are available from the National Violent Death Reporting system (contact at [nvdrs-rad@cdc.gov](mailto:nvdrs-rad@cdc.gov)) for researchers who meet the criteria for access to confidential data. Additional details are provided on the NVDRS website <https://www.cdc.gov/nvdrs/about/nvdrs-data-access.html>).”

3. **If there are no restrictions, please upload the minimal anonymized data set necessary to replicate your study findings to a stable, public repository and provide us with the relevant URLs, DOIs, or accession numbers. Please see http://www.bmj.com/content/340/bmj.c181.long for guidelines on how to de-identify and prepare clinical data for publication. For a list of recommended repositories, please see https://journals.plos.org/plosone/s/recommended-repositories. You also have the option of uploading the data as Supporting Information files, but we would recommend depositing data directly to a data repository if possible. Please update your Data Availability statement in the submission form accordingly.**

There are restrictions on our ability to share data, as described above. **We have updated our Data Availability Statement:** “Data cannot be shared publicly because of legal and ethical considerations to ensure confidentiality. Data are available from the National Violent Death Reporting system (contact at [nvdrs-rad@cdc.gov](mailto:nvdrs-rad@cdc.gov)) for researchers who meet the criteria for access to confidential data. Additional details are provided on the NVDRS website <https://www.cdc.gov/nvdrs/about/nvdrs-data-access.html>).”

4. **Please note that your Data Availability Statement is currently missing the repository name and the DOI/accession number of each dataset or a direct link to access each database. If your manuscript is accepted for publication, you will be asked to provide these details on a very short timeline. We therefore suggest that you provide this information now, though we will not hold up the peer review process if you are unable.**

**We have updated our Data Availability Statement:** “Data cannot be shared publicly because of legal and ethical considerations to ensure confidentiality. Data are available from the National Violent Death Reporting system (contact at [nvdrs-rad@cdc.gov](mailto:nvdrs-rad@cdc.gov)) for researchers who meet the criteria for access to confidential data. Additional details are provided on the NVDRS website <https://www.cdc.gov/nvdrs/about/nvdrs-data-access.html>).”

**5. Please include captions for your Supporting Information files at the end of your manuscript, and update any in-text citations to match accordingly. Please see our Supporting Information guidelines for more information: http://journals.plos.org/plosone/s/supporting-information.**

We have provided these captions at the end of the manuscript file, as requested.

We have also taken this opportunity to reorganize and streamline the supporting information (breaking each section of text and table/figure into its own attachment) to make it easier for the reader to access the corresponding information from the main text.

### **Comments from the Editor**

**1. The analysis relies heavily on the identification of PrC from case narratives, but the validation of this approach is limited. More rigorous evaluation of the PrC labeling method, such as having multiple raters independently code a larger subset of narratives, would strengthen confidence in the findings. (Also see point #5 below.)**

We agree and in response to this comment we have had multiple independent raters manually review and label PrC from the case narratives. Overall, in this revised submission, we manually labeled 250 ‘PrC-positive’ cases and 450 ‘PrC-negative’ cases to estimate Positive Predictive Value (PPV) and Negative Predictive Value (NPV), respectively. We manually reviewed more ‘PrC-negative’ than ‘PrC-positive’ cases given our expectation that the ‘true’ prevalence of PrC will be low, and therefore additional cases are needed to correctly estimate NPV. We also use a Bayesian approach to simulate the NPV using this empirical data.

We have also moved text regarding our case definition (lines 177-203) and details regarding the validation procedure (lines 204-266, including new **Figure 1**) from the **Supporting Information** which we have reorganized and broken into separate documents (S2 and S3 Appendices relate to case identification) to make them easier for the reader to access**.**

While details are provided in the text and in **S2 Appendix,** we summarize the key elements of our evaluation of the PrC labeling here:

Among the 35,861 decedents included in our analytic sample, we applied our definition of pandemic-related circumstances (PrC) to identify cases from the narrative texts, i.e., the text had to *explicitly describe that some distress, event, or precipitating behavior in the decedent’s life was connected to the onset or continuation of COVID pandemic*. This is summarized by new **Figure 1**.

- To do this, we first parsed the narratives using an initial set of key words/phrases, identified by the research team, which signified the pandemic (e.g., “covid,” “pandemic,” “lockdowns”), which identified 2,876 narratives.
- We then augmented this set of words/phrases using text analysis tools to identify additional terms that were semantically similar (e.g., “distance learning,” “shelter-in-place orders”), which identified an additional 25 narratives.
- Finally, we identified a set of exclusion phrases which conveyed information about the pandemic, in a manner that did not meet our case definition of PrC (e.g., “covid: negative,” “covid: no”).

We evaluated the performance of our case identification approach using extensive manual review by independent raters.

- To begin, three independent annotators labeled subsets of ‘PrC-positive’ and ‘PrC-negative’ cases, as identified from the key word/phrase approach described above.
- Trained annotators then independently manually reviewed 500 cases (250 PrC ‘positive’ and 250 PrC ‘negative’) to assess inter-annotator agreement and evaluate the accuracy (PPV, NPV) of this keyword/phrase approach to identifying PrC.
- Given that PrC cases are expected to be relatively infrequent, we supplemented the NPV analysis with manual review by independent raters of two additional subsamples (total n=200) of ‘PrC-negative’ cases that were selected because they were more likely to contain false-negatives (e.g., cases that had narrative texts with related key words/phrases as our inclusion terms, and cases that had features (i.e., date of death, narrative length) that were similar to the PrC-positive cases.
- Finally, we augmented this manual review by using a Bayesian model to calculate the expected distribution of the NPV of the PrC identification procedure, using the original random sample of n=250 ‘PrC-negative’ cases.
- Overall, assuming manual review of the narrative as the gold-standard, the PPV of our PrC case identification approach was 0.92 and the NPV was 0.99.

**2. While the topic modeling provides interesting insights into PrC themes, the analysis is largely descriptive. Applying additional quantitative measures, such as calculating topic prevalence by demographic group, could enable statistical comparisons and uncover meaningful patterns.**

We agree that exploring topics across demographic factors could be interesting, however our preliminary exploration of this illustrated that the only demographic factor consistently associated with the topics was *age group*, which is what is illustrated by **Figure 3** (previously **Figure 2** in the original submission).

In response to this comment, we have created a new figure (**S9 Figure**), which is a forest plot showing the relative odds of each topic as a function of *age group, sex, marital status, race,* and *educational attainment.* It shows that with few exceptions, only age group was significantly associated with the prevalence of topics. We have also added text describing the creation o**f S9 Figure** to the **Main Text** methods (lines 306-310) and have added interpretation of this figure to the **Results** (lines 365-371). Please see revised text and supplement.

**3. The time series analysis comparing 2020 to pre-pandemic years is useful but appears somewhat simplistic. Employing more sophisticated time series modeling techniques that account for seasonality, autocorrelation, and other temporal factors could provide a more nuanced understanding of pandemic-related changes in suicide rates.**

Thank you for this comment. In response we have clarified how our existing procedure accounts for seasonality and autocorrelation (lines 485-490) of the **Main Text** (reprinted below, text in italics is new):

“We conducted a time series analysis by directly standardizing the 4-week rolling averages in 2020 to the corresponding average case counts of 2017/2018. Values <1 indicate that 2020 had fewer deaths than the pre-pandemic period in that week, and values >1 indicate that 2020 had more deaths. *This procedure accounts for seasonality in the baseline period suicide risk and the effects of other temporally-varying factors by showing 2020 mortality relative to the baseline period mortality. This procedure accounts for autocorrelation by comparing 4-week moving averages rather than weekly suicide frequencies.”*

While we know that there are other approaches to account for seasonality (e.g., more sophisticated methods like ARIMA or exponential smoothing), in order to implement such approaches we would need to have more than two years of pre-pandemic data (2017-2018). While the NVDRS was instituted pre-2017, it is a system that adds more states each year and the further in time we go back, the fewer states we have that have been reporting into the NVDRS on a consistent basis (i.e., the system started in 2003 with just 5 states reporting). While we have shown that our results are not sensitive to using baselines from 2015 and 2016 (**S6 Figure**), we believe that the analysis is stronger if it includes more states.

We have also added text to the **Discussion** that addresses the challenge of examining variation in suicide mortality over the calendar year (please see revised text in lines 445-451)

**4. The conclusions drawn from the findings seem rather limited. The authors could delve deeper into the implications of their results for suicide prevention efforts during future crises. For example, what interventions might mitigate the elevated risks associated with financial strain and isolation? How can messaging be tailored to different age groups given the varying PrC themes?**

We agree that our initial manuscript did not fully engage with the implications of our findings post-2020. In response to this comment, we have substantially re-written the **Discussion** to now engage with this issue directly. Please see lines 391-410 and 411-442 in the revised manuscript, as well as our response to comment #6 below.

**5. The study relies on the assumption that pandemic-related circumstances were consistently and accurately recorded in the case narratives. If such PrC were sometimes omitted or incorrectly mentioned, this could lead to underestimation or overestimation of the prevalence and impact of PrC. A particular case of interest would be if the narratives happen to be biased based on some protected attribute (age, race, gender, etc.), so that victims of a particular class are more/less likely to be described as subject to PrC. The authors should thus discuss the potential limitations of using narrative data and how this might affect the interpretation of their findings.**

We agree that there are many biases reflected in the narratives themselves – including disparities in death investigations, in the completeness of medical examiner and law enforcement reports, and in coding by NVDRS abstractors. We have acknowledged this as a limitation in a new paragraph in the **Discussion** (please see revised text in lines 460-481).

**6. Finally, the generalizability of the findings to suicides beyond 2020 is unclear. Acknowledging the unique circumstances of the pandemic's first year and the need for ongoing research as the crisis evolves would be valuable additions to the discussion.**

We agree that our initial manuscript did not fully engage with the implications of our findings post-2020. We have substantially re-written the Discussion to now engage with this issue directly. Please see lines 411-442 and 492-510 in the revised manuscript.
