# Supplementary material for: Psychosocial and pandemic-related circumstances of suicide deaths in 2020: Evidence from the National Violent Death Reporting System
Source: PLoS One. 2024 Oct 11;19(10):e0312027. doi: 10.1371/journal.pone.0312027 (PMC11469549; doi:10.1371/journal.pone.0312027)
Supplement: Attachment — Submitted filename: COVID PLOS 1 Response to Reviewers.docx [file pone.0312027.s012.docx]

**Reviewer #1: This was a well-written, methodologically rigorous, meticulously documented and interesting paper looking at COVID-related circumstances among suicide decedents, using data from CDC’s National Violent Death Reporting System. I’d like to see the authors address the following feedback, however:**

**1. The introductory epidemiology piece needs some edits for accuracy/clarity. Suicide is currently the 11th leading cause of death, not the 12th. Numbers need to be updated using NVSS 2022 data, and there were over 46,000 deaths by suicide in the U.S. in 2022, not “almost” 46,000. Additionally, the authors often use the term “annually” when they are talking about specific years. This should be edited to refer to specific years.**

In response to this comment we have corrected these sentences using the 2022 data from NVSS/WISQARS and provided further clarification about the specific year or 2022.

**2. Consider whether reference 4 is really needed – the data it used is the same CDC data referred to in other references.**

In response to this comment we have removed references 3 and 4 since they both utilize the CDC data in WISQARS cited in reference 1.

**3. References 11 and 12 refer to the same paper. The authors do not need both.**

In response to this comment we have removed reference 12, as it is a press release of the same report referenced in 11, to avoid redundancy.

**4. The description of NVDRS narratives in the manuscript is not exactly correct. While the authors frame the narratives around the “salient circumstances” in a victim’s life at the time of death, they are really intended to tell the story of the incident. Per the NVDRS Coding Manual version 6.0:
Narrative accounts of the incident serve multiple purposes:** **To briefly summarize the incident (who, what, when, where, why)** **To provide supporting information on circumstances that the abstractor has endorsed in an incident** **To provide the context for understanding the incident** **To record information and additional details that cannot be captured elsewhere** **To facilitate data quality control checks for coding key variables

Edits are also needed on line 178, and 463-465 where this description is given again.**

In response to this comment we have modified the text in lines 90-103 (where the narratives are first introduced in the manuscript), lines 180-190 (description of the narrative texts), and lines 500-521 (discussion of limitations of these texts) to provide the clarification requested.

**5. Line 112 needs to be updated. NVDRS is soon releasing 2022 data. Edit to clarify this and that at the time of the analysis (not “currently) it included over 360,000 suicide and undetermined deaths.**

Thank you for pointing this out. We have added this clarification to line 112.

**6. Line 273: Minor typo. “Visualized” should be “visualize”**

We have made the change as indicated.

**7. The authors should explain where the employed vs. unemployed information came from.**

In response to this comment we have added the following text to Measures/Decedent Characteristics section of the Methods and S1 Appendix to explain the coding of this variable:

“Finally, because the existing NVDRS quantitative variables provide only limited detail regarding employment characteristics, we created a 6-level categorical indicator for labor force status using a keyword search of the text fields “Occupation Current Text” and “Occupation Text_DC”. This variable was coded as: industry employment, self-employment, disability, retirement, student, unemployment (includes decedents who were incarcerated), and unknown/missing.”

**8. Line 336: Appears to be missing a word**

We have corrected this error as indicated and added the word “of” in line 336.

**9. Line 341: Minor typo. Should be substance use problems, not problem.**

We have made the change as indicated.

**10. How were containment measures separated form concerns about containment measures? (Line 348)**

In response to this comment we have reconciled the text (lines 378-391) and the S6 Table to ensure that they use the same terminology when describing these aspects of infection control (we realized that the text about the containment measures and concerns was carried over from an earlier draft). We have corrected the text to reflect the final set of topics reflected in S6 Table.

The text now reads: “The 11 topics identified in the narratives with PrC fell into four broad, non-mutually-exclusive groupings: (1) Public health containment measures and related concerns [topics in this grouping: *COVID-19 testing and isolation* (prevalence: 19.9%), *COVID-19 restrictions* (prevalence: 19.3%), and *Quarantine, movement, and change of space* (prevalence: 16.9%)]; (2) Changing social and economic situations [topics in this grouping: *Job or business loss* (prevalence: 14.3%), *Unstable work, financial or family environment* (prevalence: 18.1%), and *Remote schooling and social adjustment* (prevalence: 11.2%)]; (3) Physical health problems and concerns [topics in this grouping: *COVID-19 testing and isolation* (prevalence: 19.9%) and *COVID symptoms and other health problems* (prevalence: 17.9%)]; and (4) Psychological distress and concerns [topics in this grouping: *Stress and problems* (prevalence: 11.5%), *Fear and frustration* (prevalence: 20.6%), *Mental health symptoms exacerbated by the pandemic* (prevalence: 12.7%), and *Isolation and related anxiety* (prevalence: 18.0%)]. See **S6 Table** for exemplar narrative excerpts.”

As noted in the revised text, these groupings are not mutually-exclusive (e.g., narratives could have >1 topic represented). Indeed, we acknowledge that the narratives provide only limited evidence to distinguish whether the decedent was “concerned” about the containment measures themselves vs. distressed by the impact/consequence of said measures (e.g., were they worried about the safety of masks vs. were they experience distress because their job was considered “essential”). These two scenarios are generally not distinguishable using the narrative texts, even using the topic models we employed, because of the manner in which they were written. Please also see the first paragraph of the Limitations in the Discussion.

**11. Line 355 and S11: NVDRS data sharing agreements require narrative excerpts to have details changed or be composites to protect the privacy of decedents. It is not noted in the manuscript or supplemental materials if this was done. This should be done and noted.**

In response to this comment all cited narratives have been further anonymized by removing all demographic details (they had already been shortened and potentially-identifying details removed in the prior submission). We have now added the following statement in the main manuscript and S6 Table: “In accordance with the requirements of the NVDRS data sharing agreement, all cited narratives have been modified to protect the privacy of decedents.”

**12. Line 526: NVDRS is administered by CDC and participating states.**

We have made the change as indicated.

**13. Figure 3: It is not clear why there are CIs on this figure given that these are counts.**

(Note: now that the sample selection flowchart has been moved into the main text, the figure this comment is now referring to has been renumbered as Figure 4 in the main text).

For this figure we are comparing the frequency of *topic* mentions across decedents of different age groups (rather than comparing case counts per say). We wanted to give some sense of the amount of uncertainty in assigning the 11 topics we identified. Note that we set a threshold of topic probability of >0.10 (at least 10%) for this analysis because this threshold captured most (85%) of the topics represented in the narratives and provided interpretable, relatively homogenous topic groupings (see S6 Table). However, other investigators could have chosen a different topic probability threshold and thus there is uncertainty in the topic assignment.

Therefore, to generate the error bars in Figure 4, we assume that the number of deaths with PrC is fixed and then model uncertainty in the presence (and assignment) of the 11 topics to the PrC narrative texts using a binomial distribution. Using this approach, comparing two age groups is equivalent to a two-sample proportion test and error bars are taken from a one-sample proportion test. We feel the error bars emphasize the uncertainty in the process of assigning topics, which is consistent with our overall conservative approach to this analysis.

We have now added this text to S5 Appendix (section 6) and reference it in the caption of Figure 4.

**14. S1: The authors state: “For example, a recent validation study found that NVDRS circumstance variables missed to indicate about a half of cases with known mental health and substance use problems.1 However, the reference called out by the superscript is not given. This is a strong critique of the validity of NVDRS circumstances and needs corroboration. Thank you for the opportunity to review this work.**

Thank you for catching this omission. In response, we have now added the relevant citation and corrected the text in the S1 Appendix: “For example, a recent validation study of Oregon NVDRS behavioral variables found that these circumstance variables missed to indicate about a half of cases with known mental health and substance use problems.(1)”

Citation:

1. Carlson, K. F., Gilbert, T. A., DeFrancesco, S., Wright, D. A., Shen, X., & Cook, L. J. (2022). Accuracy of behavioral health variables in Oregon national violent death reporting system data: a linked cohort study. Injury epidemiology, 9(1), 29.

**Reviewer #2: The present manuscript addresses pandemic-related circumstances among suicide victims during 2020, taking into account a range of pre-pandemic data, sociodemographic and psychosocial factors. The study is relevant given the uncertainty about the relationship between COVID-19 and suicide deaths. The introduction is well-written, the relevance of the topic is clear, and the line of argumentation is coherent. In the methods section, a detailed description of the data source is provided. Justification for the exclusion of the data collected in 2019 is also sensemaking.
The results are clearly presented, and the supplementary files are comprehensive, providing helpful information about the procedure. Furthermore, the discussion is well-structured, it contains many important and interesting points. Overall, the manuscript reads well and I have only identified a few minor issues. These are presented below:

1. Abstract: In the last sentence (Conclusion), add „and“ before „can inform“.**

We have made the change as indicated.

**2. Introduction: Line 86: Add „of“ before „the potential contribution“.**

We have made the change as indicated.

**3. Materials and Methods: Line 138: Replace „restrict“ with „restricted“, given that the rest of the methods section is written in past simple tense.**

We have made the change as indicated.

**4. Line 199: It would be good if you could provide an additional explanation on why being tested positive for COVID-19 was not coded as pandemic-related circumstance. I think that a COVID-19 infection at the time of death is a relevant factor, so I would appreciate if you could provide a justification for your decision in the revision.**

Thank you for this note. As we describe in the ‘Methods’ under the “Case definition of ‘pandemic-related circumstances (PrC)’ from the narrative texts,” we adopted a conservative approach toward determining which cases should be included in the PrC category. That is, we ascertained a case as a PrC only if the narrative ‘*explicitly describe that some distress, event, or precipitating behavior in the decedent’s life was connected to the onset or continuation of COVID pandemic’.* In other words, a narrative had to indicate that COVID-related circumstances (e.g., being infected with the virus) were among the contributing factors to the death. This decision was consistent with the NVDRS case definition for salient circumstances (e.g., ‘disaster exposure’ variable that includes exposure to COVID-19 among other man-made or natural disasters).

When a narrative simply mentioned COVID testing or a testing result (which would often be recorded in the text as simply “COVID: positive” or “COVID: negative” without any additional context), there was no way for us to know whether the decedent was aware that they had COVID at the time of death, whether the positive test result was relevant to their death, *or even whether the testing occurred post-mortem (and therefore could not have reasonably considered as a contributing circumstance)*. Particularly early in the pandemic it was unclear whether routine COVID testing of suicide/undetermined deaths decedents was occurring or if reporting COVID testing results post-mortem was a standard practice across all states in the coroners/medical examiner reports. In sum, in 2020 the narratives were inconsistent in whether and how they described COVID test results (in isolation), particularly as it relates to the notion of the test result being a “contributing circumstance.”

However, we also note that we only excluded cases indicating “COVID: positive” (for example) if this was the only pandemic-related factor mentioned in that narrative. For instance, if a narrative stated that “after testing positive for covid-19, the victim self-quarantined and had been struggling with anxiety due to the diagnosis,” this cases was labeled as being PrC-positive, since it is explicitly stated that the decedent was aware they had COVID and there is sufficient context to indicate it was a contributing circumstance to the death in the manner that other circumstance variables in this data are coded.

In response to this comment we have revised text lines 192-205 to provide additional justification for our case identification process as it relates to COVID testing/results reported in the narrative.

**5. Discussion: I suggest rephrasing the third sentence as follows: „Victims of suicide deaths with PrC tended to be older and more educated relative to those whose deaths did not include PrC“.**

In response to this comment we have revised the text as follows: “Suicide decedents with PrC tended to be older and more educated relative to those whose narratives did not include PrC.”

**6. Line 453: The sentence „These findings can inform future efforts to explore these questions.“ is unclear. Please rephrase it for comprehensibility.**

In response to this comment we have revised this line to the following: “By providing a comprehensive characterization of suicide mortality over the first year of the pandemic, these findings provide an empirical foundation from which future work can explore the impact of social, economic, and political factors on suicide mortality, particularly as the pandemic has moved beyond this emergency phase.”

**Reviewer #3: This is a very interesting study and the work is well-presented. The authors made substantive changes to address the previous reviewers' comments. This work will add very important contributions to the literature. The authors utilized a novel approach to address their study questions. I recommend acceptance for publication. However, I think this manuscript would be strengthened by MINOR revision early in the manuscript to better describes NVDRS and its data and limitations. This is important because understanding NVDRS is central to contextualizing the analyses and findings. Important clarifications/additions are those that are preceded by ** in the list below and summarized here: NVDRS did not include all states until recently, all regions of participating states may not be included, narrative data is abstracted from CME/LE reports but the information contained within those CME/LE source documents is inconsistent and non-standardized, abstractors have a lot of influence over the narrative data and when reports provide different or conflicting information abstractors record what they believe to be the most accurate narrative. A few proofreading edits are also noted.**
 **1. Background. Lines 77-80: This sentence references “numerous surveys” and “several reports” but only two citations are provided. Modify sentence or include more supporting citations.**

We agree with this suggestion and have provided additional citations to support our claims.

**2. Line 86: a word or two is likely missing from this sentence**

Thank you for catching this. We have updated the sentence appropriately.

**3. **Line 95: Re: “these narratives collected information about pandemic-related circumstances” – Might be beneficial to clarify that this information was included in NVDRS only if it was in the CME or LE reports, but that inclusion in those (CME/LE) reports is inconsistent and non-standardized.**

In response to this comment we have made the following changes to the Introduction about the narratives (lines 90-100):

“Each observation has qualitative text fields, called *case narratives,* which describe the contextual details of the incident as well as the events that preceded it, as that information is documented in the CME and LE reports. These narratives often contain information regarding circumstances in the decedent’s life that are salient to their death including psychosocial factors such as recent difficulties in relationships, work, or school, as well as mental and physical health problems. In 2020, abstractors began including information in these narratives about pandemic-related circumstances (e.g., concerns about infection, job losses due to workplace closures)(15). The contextual details of the case narratives make the NVDRS one of the only datasets that can examine correlates of suicide mortality in the context of COVID-19 pandemic on an individual level using data that is collected at the population scale.(13,16)”

In response to this and other reviewer comments, we have also expanded the text addressing inconsistent reporting in the narratives, particularly regarding COVID-19 in the Methods section (lines 192-204) as well as overall inconsistency in the Discussion (lines 510-517).

**4. Data Source: **Lines 112-114: This sentence may be a little misleading. Data is not available for all 50 states, DC, and PR dating back to 2003. NVDRS was implemented in 2003 with 5 states participating, and since then an increasing number of states contribute data each year. I don’t believe NVDRS included all 50 states until 2019. Even when a state is represented in the data, the reporting may only include a portion of the counties in the state.**

We agree with this point and have clarified the following text: “The NVDRS was implemented in 2003 with five participating states, and since then an increasing number of states contributed their annual data. At the time of analysis, the NVDRS currently includes over 360,000 suicides and undetermined deaths (henceforth “suicides”) from all 50 states, District of Columbia, and Puerto Rico, from 2003 to 2020.”

**5. Line 113: (henceforth “suicides”) -I’m interpreting this as meaning for the purpose of this dataset you are referring to both suicides and undetermined manner of death cases as “suicides”. If so, this henceforth statement might be better positioned after your explanation in Lines 120-122 on why you’ve included undetermined deaths.**

We have corrected the text to reflect this suggestion.

**6. Line 163: Any vs none – Please clarify that the none are derived from the no/not available/unknown code, which means the “none” category is a mix of cases with none and not available/unknown. I think this is important point because of information you highlight in the S1 appendix: “NVDRS circumstance variables encode valuable information, however these variables are often limited by data availability and structural changes in data processing. For example, a recent validation study found that NVDRS circumstance variables missed to indicate about a half of cases with known mental health and substance use problems.”**

We agree with this suggestion and have corrected the text accordingly.

**7. Line 170-172: 150-300 words, the case inclusion strategy was previously described to include cases with narratives >= 35 characters, how does this align with the 150 word lower limit? These seem inconsistent. Please clarify this discrepancy.**

Thank you for the opportunity to clarify this. This text refers to an *approximation* of the word counts as described in prior analyses of the NVDRS narratives by our and other investigator groups.

In response to this comment, we have revised the text to state the word count of the actual narratives we used in our sample, not just the approximations from prior work. The paragraph describes average number of each type of the narrative as follows:

“The CME and LE narratives are paragraphs of free text written by abstractors from original source documents to provide contextual details of the incident as well as salient circumstances of the decedent’s life at the time of death (15). These texts are informational summaries of all data sources, which typically reflect the quantitative variables in the dataset, and include additional contextual information not encoded in the quantitative data. In the 2020 data used in this analysis, the average word count of CME and LE narratives were 152 words (SD: 107) and 119 words (SD: 117), respectively.”

**8. Measures: **Qualitative narrative text: Data abstractors have a lot of influence over the narrative data. For example: the NVDRS coding manual (NATIONAL VIOLENT DEATH REPORTING SYSTEM WEB CODING MANUAL VERSION 6.0* (**[**cdc.gov**](http://cdc.gov/)**)) provides the following instruction on page 25: “Sometimes information across or within C/ME and LE reports may provide different or conflicting information…If multiple LE reports conflict about the narrative details, record what you believe to be the most accurate narrative. Do the same for multiple C/ME reports.”**

In response to this comment we have substantially revised the description of the narrative (see lines 182-205) to reflect this point. Please also see our responses above and our response to Reviewer #2 (comment #4).

We have expanded our explanation of the limitations of the narratives Discussion (lines 510-517):

“This analysis relies on the assumption that pandemic-related circumstances, as defined in this study, were consistently and accurately recorded in the case narratives. However, due to biases in the data generation process, including in the processes of legal investigations and writing of the narratives by abstractors, information regarding pandemic-related factors may be incomplete from the narratives of some groups.(16,39)”

**9. Analysis: Line 273: “visualized” should be “visualize”**

We have made the change as indicated.

**10. Line 301: the second “an” should be “a”**

We have made the change as indicated.

**11. Discussion: Line 408: the word “did” is likely missing**

We have made the change as indicated.

**12. Please number the tables, figures, and supplemental material sequentially in the order they appear in the manuscript**

We have renumbered tables/figures and supplemental materials so they are now sequential in the order they appear, as requested. We also moved the analytic sample flowchart into the main text as we feel this is critical information for the reader.
